# Hand hygiene compliance at neonate intensive care unit in a Brazilian hospital

**DOI:** 10.1186/1753-6561-5-S6-P128

**Published:** 2011-06-29

**Authors:** LR Barbosa, MAS Rego, AM Santos, S Colacioppo

**Affiliations:** 1 Scientific, GOJO América Latina, São Paulo, Brazil; 2 Scientific, Universidade Federal de Minas Gerais, Belo Horizonte, Brazil; 3 Scientific, Rumel Santos Healthcare Training and Consulting, Calgary, Canada; 4 Scientific, Universidade de São Paulo, São Paulo, Brazil

## Introduction / objectives

Several factors influence compliance to hand hygiene (HH) and different variables can be evaluated to improve quality of care assessment, incentive for performance improvement, outbreak investigation and infrastructure design.

## Objective

To describe the general compliance to HH associated with variablesof interest in a study of direct observation (DO) in a NeonateÂ Intensive Care Unit (NICU) in a University Public Hospital.

## Methods

DO was performed by 10 observers. Variables were # beds, # health care workers, professional category, type of opportunity, day of week, time of the day and product used. Statistical analysis used software Stata and SPSS for Windows.

## Results

A total of 7,324 opportunities for HH were identified during 255 1 hour DO periods from December 2008 to March 2009. Overall compliance rate 50.2%. 69.5% for medical doctor (MD), 60.8% for other professionals (OP), 48.7% for nurses (RN) representing respectively 10.8%, 12% and 70.3% of all opportunities. Highest compliance was before aseptic procedure for MD and OP (100% for both) and after touching patient surroundings for RN (73.1%). Lowest compliance was after patient contact for all categories: 52% (MD), 42.7% (OP) and 36.7% (RN). 55% of HH were performed with soap and water, 23.1% with alcohol-based hand rub (ABHR) and 21.9% with soap and water followed by ABHR.

## Conclusion

DO study results indicated that the NICU did not follow CDC 2002 guideline recommendation for HH. Compliance may be improved by focusing on the opportunities to perform HH using ABHR products.

## Disclosure of interest

L. Barbosa Employee of Luciana Barbosa is an employee of GOJO América Latina, M. A. Rego: None declared, A. Santos: None declared, S. Colacioppo: None declared.

